# Spatial and temporal niche overlap of aardwolves and aardvarks in Serengeti National Park, Tanzania

**DOI:** 10.1002/ece3.10718

**Published:** 2023-11-07

**Authors:** Merijn van den Bosch, Kenneth F. Kellner, Imani Mkasanga, Stanslaus B. Mwampeta, Robert Fyumagwa, Mariela G. Gantchoff, Brent R. Patterson, Jerrold L. Belant

**Affiliations:** ^1^ Department of Fisheries and Wildlife Michigan State University East Lansing Michigan USA; ^2^ Wildlife Conservation Initiative Arusha Tanzania; ^3^ Department of Biology University of Dayton Dayton Ohio USA; ^4^ Ontario Ministry of Natural Resources Trent University Peterborough Ontario Canada

**Keywords:** camera trap, commensalism, occupancy model, *Orycteropus afer*, *Proteles cristata*, spatiotemporal niche

## Abstract

Species interactions can influence species distributions, but mechanisms mitigating competition or facilitating positive interactions between ecologically similar species are often poorly understood. Aardwolves (*Proteles cristata*) and aardvarks (*Orycteropus afer*) are nocturnal, insectivorous mammals that co‐occur in eastern and southern Africa, and knowledge of these species is largely limited to their nutritional biology. We used aardwolf and aardvark detections from 105 remote cameras during 2016–2018 to assess their spatial and temporal niche overlap in the grasslands of Serengeti National Park, Tanzania. Using a multispecies occupancy model, we identified a positive interaction between occupancy probabilities for aardwolves and aardvarks. Slope, proportion of grassland and termite mound density did not affect the occupancy probabilities of either species. The probability of aardwolf, but not aardvark, occupancy increased with distance to permanent water sources, which may relate to predation risk avoidance. Diel activity overlap between aardwolves and aardvarks was high during wet and dry seasons, with both species being largely nocturnal. Aardwolves and aardvarks have an important ecological role as termite consumers, and aardvarks are suggested to be ecosystem engineers. Our results contribute to a better understanding of the spatial and temporal niche of insectivores like aardwolves and aardvarks, suggesting high spatial and temporal niche overlap in which commensalism occurs, whereby aardwolves benefit from aardvark presence through increased food accessibility.

## Graphical abstract

We investigated the spatial and temporal niche of two insectivorous mammals, aardwolves (*Proteles cristata*) and aardvarks (*Orycteropus afer*) and quantified niche interactions between them in the grasslands of Serengeti National Park, Tanzania. We provide evidence of spatial and temporal niche overlap between aardwolves and aardvarks.

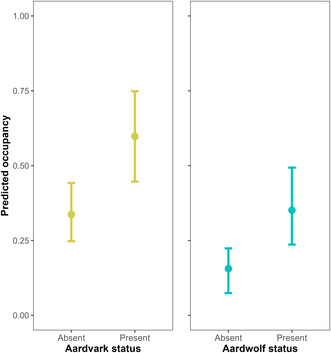

## 1 INTRODUCTION

Species distributions are determined by environmental conditions and interspecific interactions (Wiens, 2011). Ecological niche overlap between sympatric species can cause competition, resulting in reduced abundance or exclusion of weaker competitors (Creel & Creel, 1996; Miquelle et al., 2005). Competition can be avoided through niche partitioning (Schoener, 1974) which facilitates species diversity (Chesson, 2000; Levine & HilleRisLambers, 2009). Alternatively, sympatric species with high niche overlap may interact non‐competitively. Two species can benefit from a symbiotic interaction through mutualism, while commensalism occurs when one species benefits and the other is unaffected (Mathis & Bronstein, 2020). Understanding species interactions can help conserve ecological communities because they influence species distributions (HilleRisLambers et al., 2012; Wisz et al., 2013) alongside other factors such as anthropogenic landscape change (Broennimann et al., 2012).

Behavioural and morphological similarities between species can cause niche overlap (Brown & Wilson, 1956; Dayan & Simberloff, 2005), with behavioural niche overlap occurring along the axes of space, time and diet (Schoener, 1974). Dietary niche overlap can be mediated through dietary differentiation, which can facilitate coexistence when species specialize in different foods (Emrich et al., 2014; Ferretti et al., 2020; Kartzinel et al., 2015). Symbiotic relationships inherently require spatial niche overlap between species, while competing species can reduce the frequency and intensity of overlap through spatial partitioning (Rodriguez Curras et al., 2022; Sollmann et al., 2012). Similarly, symbiotic relationships may require temporal niche overlap, while competing species may display temporal niche partitioning through differences in foraging time, frequency or effort (Dröge et al., 2017; Kronfeld‐Schor & Dayan, 2003).

Niche overlap can vary spatiotemporally (Wiens, 1989), for example, when dietary niche overlap varies with temporal (Porter et al., 2022) or spatial (Hasui et al., 2009) differences in food availability, or when species display decreased spatial overlap during the reproductive season (McConnell et al., 2008). For example, arctic foxes (*Vulpes lagopus*) may avoid competition with red foxes (*V. vulpes*) during the reproductive season by denning at higher elevations (Tannerfeldt et al., 2002). Risk avoidance can also influence the extent of niche overlap between species, such as when avoidance of human disturbance increases spatial and temporal niche overlap between interacting species (Sévêque et al., 2020). Bobcats (*Lynx rufus*) and pumas (*Puma concolor*) had increased overlap in activity patterns in areas with more human disturbance (Lewis et al., 2015). Similarly, predator avoidance of sympatric species can influence niche overlap as refuge from predation is shared or partitioned in space and time (Holt, 1984; Sommers & Chesson, 2019).

Aardwolves (*Proteles cristata*) and aardvarks (*Orycteropus afer*) are nocturnal mammals co‐occurring in eastern and southern Africa (Kingdon, 2015). Aardvarks have a diverse diet of termites and ants that varies geographically and seasonally (Taylor, 2013), while aardwolves depend on grass‐harvesting termites of the genus *Trinervitermes* throughout their range (De Vries et al., 2011; Kruuk & Sands, 1972). Different degrees of dietary specialism may limit spatial niche overlap of aardwolves and aardvarks, as *Trinervitermes* occur only in open habitats such as grasslands (Anderson, 2013; Kruuk & Sands, 1972), while aardvark habitat use is more diverse (Kingdon, 2015; Melton, 1976). Termite activity may be higher on slopes, attracting termite predators, so spatial overlap of aardwolves and aardvarks may increase with slope (Freymann et al., 2010; Sarcinelli et al., 2009). Areas with high large carnivore activity could increase spatial overlap between aardwolves and aardvarks in other areas as they have common predators (Anderson, 2013; Mills, 1984; Taylor, 2013), and aardvarks might avoid areas near water to avoid large carnivores (Epps et al., 2021). A commensal relationship has been suggested whereby aardwolves increase spatial overlap with aardvarks during seasons of lower food availability, to benefit from increased termite availability at locations where aardvarks excavate termite mounds (Taylor & Skinner, 2000). In the Serengeti grasslands, there is lower food availability for aardwolves during the wet season (Kruuk & Sands, 1972), which may coincide with a switch in primary prey of aardvarks from ants to termites (Melton, 1976). Additionally, seasonal variability in termite nocturnality may increase diurnal activity of aardwolves (Richardson, 1987) and aardvarks (Weyer et al., 2020), and there are no reported indications of temporal niche partitioning related to their diel activity.

We used remote camera data to quantify spatial overlap (occupancy) and temporal overlap (diel activity) of aardvarks and aardwolves in southeastern Serengeti National Park, Tanzania. We predicted a positive correlation between the occupancy probabilities of the two species. We predicted that occupancy for both species would be positively associated with termite mound density and that for aardwolves only, this effect would be stronger in the wet season. Furthermore, we predicted occupancy probability for aardvarks and aardwolves would be positively associated with increasing slope and distance to water, and with proportional grass cover (as a proxy for grassland habitat) for aardwolves only. We predicted a positive correlation between the occupancy probabilities of the two species and similar diel activity patterns whereby both species would display strong nocturnality.

## 2 METHODOLOGY

### 2.1 Study area

**FIGURE 1:**
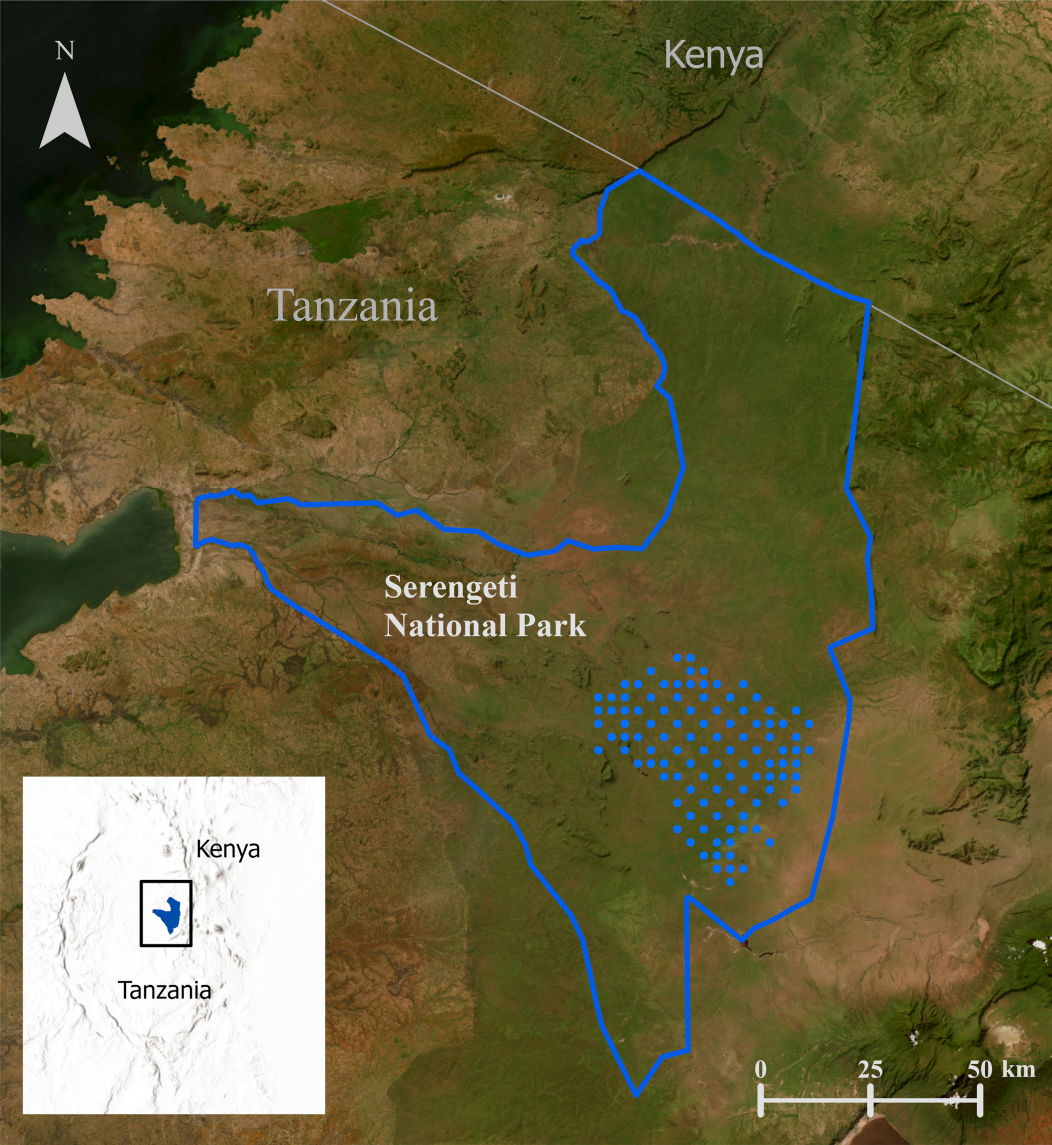
Camera locations (blue circles) to assess occurrences of aardvark (*Orycteropus after*) and aardwolf (*Proteles cristata*), Serengeti National Park (blue outline), Tanzania, 8 September 2016–30 June 2018. Map was created using the 2022 World Imagery Basemap in ESRI ArcGIS Pro Version 3.0.

We conducted the study in a 1533 km^2^ area of southeastern Serengeti National Park, Tanzania (Figure 1), which consisted primarily of grassland (89%), shrubland (7%) and woodland (2%) (Buchorn et al., 2020). Elevations are 1484–1859 m above sea level, with higher elevations in the eastern half of the study area (NASA et al., 2018). The climate is warm with stable temperatures averaging around 21°C (Metzger et al., 2015), and most precipitation occurs during November–May (Norton‐Griffiths et al., 1975). Serengeti National Park has several large carnivores that prey on aardwolves and aardvarks, including spotted hyena (*Crocuta crocuta*), lion (*Panthera leo*), leopard (*P. pardus*) and cheetah (*Acinonyx jubatus*) (Anderson, 2013; Craft et al., 2015; Taylor, 2013).

### 2.2 Data collection and processing

We collected data from August 2016 to June 2018 using 105 remote cameras (Stealth Cam, model N45NG; Irving, Texas, USA). Nearest distance between cameras was 3000 m for 63 cameras and 4225 m for 43 cameras (Figure 1). We attached cameras to metal stakes 50‐cm above ground and cleared vegetation in front of cameras every 6 weeks, with cameras programmed to record 3‐image bursts at each detection with a 30‐s delay. Because of staggered camera installations, we extracted data for a period of 70 consecutive days from each camera (hereafter a ‘camera‐period’) from 26 August 2016 to 1 January 2018, and a second 70‐day camera‐period for 77 cameras from 22 January 2017 to 30 June 2018. Overlap between the two periods of data collection occurred due to staggered camera installation. Most (95%) data were obtained from September 2016 to February 2018. Each 70‐day camera‐period was associated with a season (wet season, November–May; dry season, June–October). For 77 camera‐periods that overlapped two seasons we split the data by season into two separate, shorter camera‐periods each entirely with one season.

### 2.3 Covariates

We collected environmental covariates in a 50‐m radius around each camera. We used proportional grass cover from the 2017 Copernicus Global Land Cover dataset (range = 0–100, 100‐m resolution; Buchorn et al., 2020) as a proxy for habitat type, with lower proportional grass cover implying higher proportional shrubland or woodland. We derived the slope from the ASTER Global Digital Elevation Model v3 (30‐m resolution; NASA et al., 2018). We derived the distance from each camera to the nearest water feature using the Serengeti GIS and Data Centre (30‐m resolution; Maliti et al., 2008). We used only year‐round water sources for consistency between seasons (Schooler et al., 2022). We counted active termite mounds based on termite presence and signs of recent activity in a 50‐m radius around each camera. We proportionally averaged covariate values where multiple values occurred within a 50‐m radius. We scaled covariates before analysis by subtracting the mean from each value and dividing this by the standard deviation to facilitate comparison of parameter estimates. We used Pearson's correlations to test for multicollinearity (|*r*| > 0.7), retaining the covariate from pairwise correlations considered most ecologically relevant (Dormann et al., 2013).

### 2.4 Occupancy models

We used single‐season multispecies occupancy models to estimate the effect of environmental covariates on the occupancy of each species (MacKenzie et al., 2002) and to determine interactions in occupancy probability between the two species (Rota et al., 2016). To allow for covariate effects that vary between wet and dry seasons, we treated camera‐periods (defined above; data from a given camera during a given time period of ≤70 consecutive days, separated by wet and dry season) as separate sites for the purposes of the occupancy model (a ‘stacking’ approach; Kéry & Royle, 2020). Preliminary analysis showed a high proportion of non‐detections led to numerical estimation and optimization problems (Steenweg et al., 2016; Tobler et al., 2015), so we pooled data points into 5‐day detection periods whereby every 5 days of data collection were grouped into one sampling occasion (i.e. ≤ 70 daily sampling occasions became ≤14 5‐day sampling occasions).

Our occupancy models assumed occupancy was spatially and temporally constant for a site at a site throughout a ≤70‐day sampling period (Rota et al., 2009). We considered this reasonable, as aardwolves and aardvarks occupy small territories (1–4 km^2^) relative to the spacing between cameras (3–4 km) in our study area, with little to no territory overlap between conspecifics (Anderson, 2013; Bothma & Walker, 1999; Taylor, 2013; Van Aarde et al., 1992). Furthermore, aardwolves and aardvarks are considered long‐lived species, based on lifespans in captivity exceeding 20 years (Anderson, 2013; Taylor, 2013).

We defined 10 candidate occupancy models based on our predictions which were implemented in R (v4.2.2, R Core team, 2022) using the *unmarked* package (Fiske & Chandler, 2011; Kellner et al., 2023). Models included covariates potentially affecting single‐species occupancy probabilities or species interaction: distance to water, proportional grass cover, termite mound density, slope and interactions with seasonality (Table 1). We included the number of trees present at camera sites as a detection covariate in all models. We included no other detection covariates because we cleared vegetation around cameras every 6 weeks, oriented cameras to avoid obstructed views and did not orient cameras toward roads, so we expected no other influences on detectability. A covariate was considered to have a significant effect if the 95% confidence interval did not overlap zero. We ranked models using Akaike's Information Criterion (AIC; Arnold, 2010) and selected a final model from candidate models based on the lowest AIC, or the competing model (ΔAIC <2) with fewer terms (Burnham et al., 2011). We also tested the out‐of‐sample predictive performance of the candidate models with *k*‐fold cross‐validation using an approach similar to Broms et al. (2016). For each model, we divided the data into *k* = 10 folds, then re‐fit the candidate models 10 times. For each re‐fit of *k*, one fold was held out as testing data and the remaining nine were used as training data. We then calculated the total log‐likelihood *ll*_*k*_ of the held‐out testing data in fold *k*. Finally, we calculated the total model deviance as $-2*\sum_{k=1}^{10}{\mathrm{ll}}_{k}$. We assessed goodness‐of‐fit for our top model with a parametric bootstrap (MacKenzie & Bailey, 2004). Using the *parboot* function in the *unmarked* package, we simulated 1000 datasets from the model and calculated the sum of squared errors (SSE) for each dataset. If the SSE from the real dataset fell within the distribution of SSEs from simulated datasets, we concluded the model fit the data reasonably well.

**TABLE 1:** Multispecies occupancy models for aardwolf (*Proteles cristata*) and aardvark (*Orycteropus after*), Serengeti National Park, Tanzania, 26 August 2016–30 June 2018.

Model	*K*	AIC	ΔAIC	Deviance
*Predator avoidance*				
Single species: Water	9	2153.54	0	2159.53
Species interaction: No covariates				
Species detection: Trees				
*Predator avoidance (inc. species interaction)*				
Single species: Water	10	2155.30	1.76	2180.89
Species interaction: Water				
Species detection: Trees				
*Habitat type*				
Single species: Grass	9	2158.80	5.26	2172.29
Species interaction: No covariates				
Species detection: Trees				
*Habitat type* (*inc. species interaction*)				
Single species: Grass	10	2160.66	7.13	2163.69
Species interaction: Grass species detection: Trees				
*Termite availability*				
Single species: Termite + Slope	11	2165.96	12.43	2173.32
Species interaction: No covariates				
Species detection: Trees				
*Global*				
Single species: Water + Slope + Grass + Termite + (Season: Termite) + (Season: Slope)	21	2167.50	13.97	2179.02
Species interaction: No covariates				
Species detection: Trees				
*Null*				
Single species: No covariates	5	2167.52	13.99	2176.34
Species interaction: No covariates				
Species detection: No covariates				
*Termite availability* (*inc. species interaction*)				
Single species: Termite + Slope	13	2168.95	15.42	2169.19
Species interaction: Termite + Slope				
Species detection: Trees				
*Seasonal termite availability*				
Single species: (Season + Termite) + (Season + Slope)	15	2173.23	19.69	2187.49
Species interaction: No covariates				
Species detection: Trees				
*Seasonal termite availability (inc. species interaction)*				
Single species: (Season + Termite) + (Season + Slope)	16	2174.91	21.37	2221.92
Species interaction: Season				
Species detection: Trees				

*Note*: Models included effects of distance to water (Water), proportional grass cover (Grass), termite mound density (Termite), slope (Slope) and interactions with season (Season), on first‐ and second‐order occupancy. Number of trees (Trees) present was used as a detection covariate.

Abbreviations: AIC, Akaike's Information Criterion; Deviance, total model deviance based on *k*‐fold cross‐validation; *K*, number of parameters; ΔAIC, AIC difference from the top model.

### 2.5 Diel activity

We assessed temporal niche partitioning in aardwolves and aardvarks by estimating overlap in diel activity using the package *overlap* (v0.3.433, Meredith & Ridout, 2018) in R. We created seasonal models to assess differences in diel activity and overlap between aardwolves and aardvarks, using a coefficient of overlap based on probability density functions. We filtered observations to a maximum of one hourly observation per species for each camera to avoid data clustering (Clauss et al., 2021). We used the estimator Δ_4_, appropriate when the least‐detected species has >75 observations and calculated 95% confidence intervals of overlap estimates by bootstrapping 1000 generated samples (Meredith & Ridout, 2018). We concluded differences in overlap estimates of diel activity between aardwolves and aardvarks when confidence intervals did not overlap.

## 3 RESULTS

Using 105 camera locations totaling 12,505 camera days. Aardwolves and aardvarks were detected on 264 and 121 days, respectively. Across the study duration, aardwolves were detected at 71 camera locations (naïve occupancy = 0.68), aardvarks at 35 locations (naïve occupancy = 0.33) and 25 locations had at least one detection of both species. Of a maximum of 14 sampling occasions per camera‐period, the range of sampling occasions across sites was 10–14 (median = 10). Proportional grass cover across locations was 44–80% (median = 63%), distance to water was 25–13,108 m (median = 1842 m), slope was 1.2–27.5% (median = 4.2%) and the number of active termite mounds was 0–12 (median = 1); 65 of 105 camera locations had at least one mound.

### 3.1 Occupancy models

**FIGURE 2:**
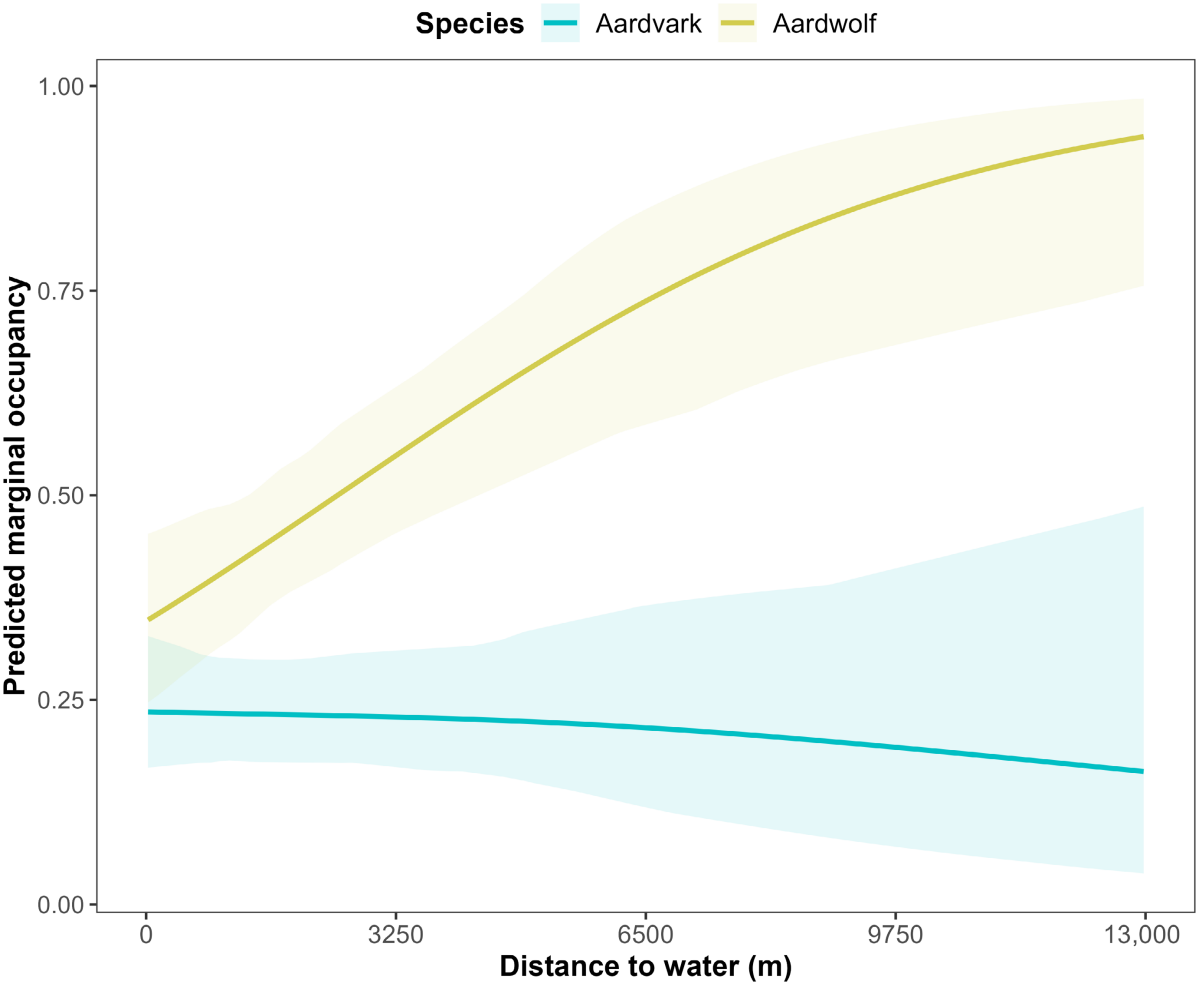
Predicted occupancy probabilities for aardvark (*Orycteropus afer*) and aardwolf (*Proteles cristata*) in relation to distance to permanent water, Serengeti National Park, Tanzania, 26 August 2016–30 June 2018. Estimates and 95% confidence intervals were derived from the top‐ranked multispecies occupancy model.

**FIGURE 3:**
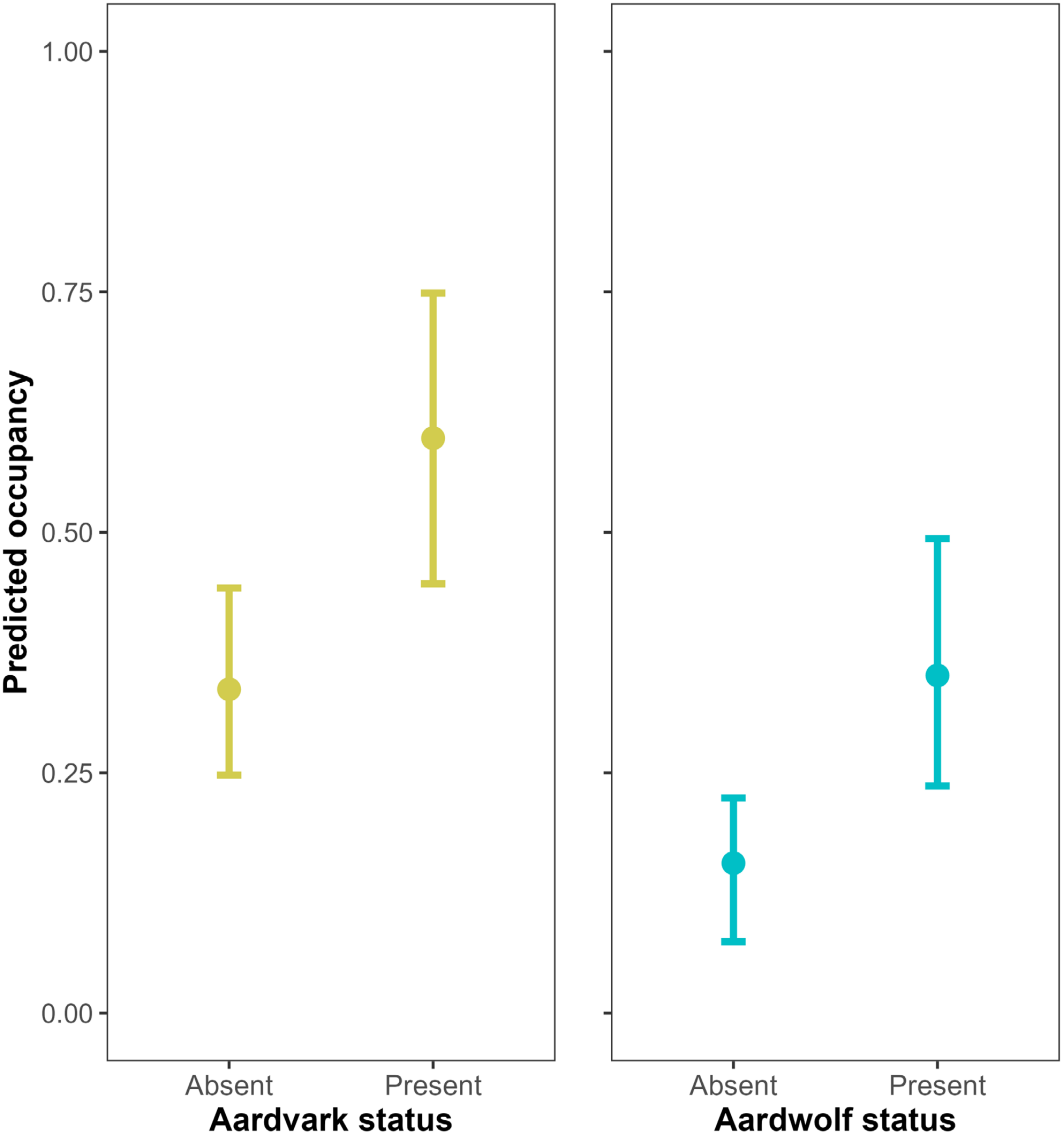
Occupancy probabilities for aardwolf (*Proteles cristata*) based on aardvark (*Orycteropus afer*) presence (left panel), and occupancy probabilities for aardvark based on aardwolf presence (right panel), Serengeti National Park, Tanzania, 26 August 2016–30 June 2018. Estimates and 95% confidence intervals were derived from the top‐ranked multispecies occupancy model.

No covariates were omitted from the analysis due to multicollinearity (all |*r*| ≤ 0.55). Occupancy for aardwolves was positively related to distance to water (CI = 0.222–0.984; Figure 2), but we found no effect of slope, proportional grass cover or termite mound density on aardwolf occupancy probability (Table 2). For aardvarks, no covariates had a significant effect on occupancy probability (Table 1). There were no differences in covariate relationships between seasons for either species. Our top‐ranked model indicated a positive interaction between aardwolf and aardvark occupancy probabilities (CI = 0.227–1.936; Table 2), suggesting a positive relationship between aardwolf occupancy probability and aardvark presence, and between aardvark occupancy probability and aardwolf presence (Figure 3). We found no effect of covariates on the interaction of aardwolf and aardvark occupancy probabilities. The number of trees at camera sites negatively influenced the detection of aardwolves only (CI = −0.868– −0.075, Table 2), though this did not notably affect relationships between our covariates and species occupancy or the interaction between species occupancies. Our top model fits the data reasonably well based on the parametric bootstrap. Deviance estimates based on *k*‐fold cross‐validation corresponded closely with AIC scores (Table 1).

**TABLE 2:** Parameter estimates, standard errors and 95% confidence intervals (CI) for the top‐ranked multispecies occupancy model for aardwolf (*Proteles cristata*) and aardvark (*Orycteropus after*), Serengeti National Park, Tanzania, 26 August 2016–30 June 2018.

Parameter	Estimate	SE	CI
*Aardwolf occupancy*			
Intercept	−0.273	0.195	−0.698 to 0.052
Distance to water	0.563	0.192	0.222 to 0.984
*Aardvark occupancy*			
Intercept	−1.809	0.334	−2.477 to −1.203
Distance to water	−0.168	0.180	−0.506 to 0.197
*Aardwolf‐Aardvark occupancy interaction*			
Intercept	1.074	0.445	0.227 to 1.936
*Aardwolf detection*			
Intercept	−1.640	0.098	−1.831 to −1.448
Number of trees	−0.471	0.202	−0.868 to −0.075
*Aardvark detection*			
Intercept	−1.718	0.146	−2.004 to 1.433
Number of trees	−0.394	0.273	−0.929 to 0.142

*Note*: The top‐ranked model included distance to water on first‐order occupancy and a second‐order species interaction term. The intercept for the interaction term had a positive, non‐overlapping confidence interval with zero (0.227–1.936), indicating a significant relationship between aardwolf and aardvark occupancy.

### 3.2 Diel activity

**FIGURE 4:**
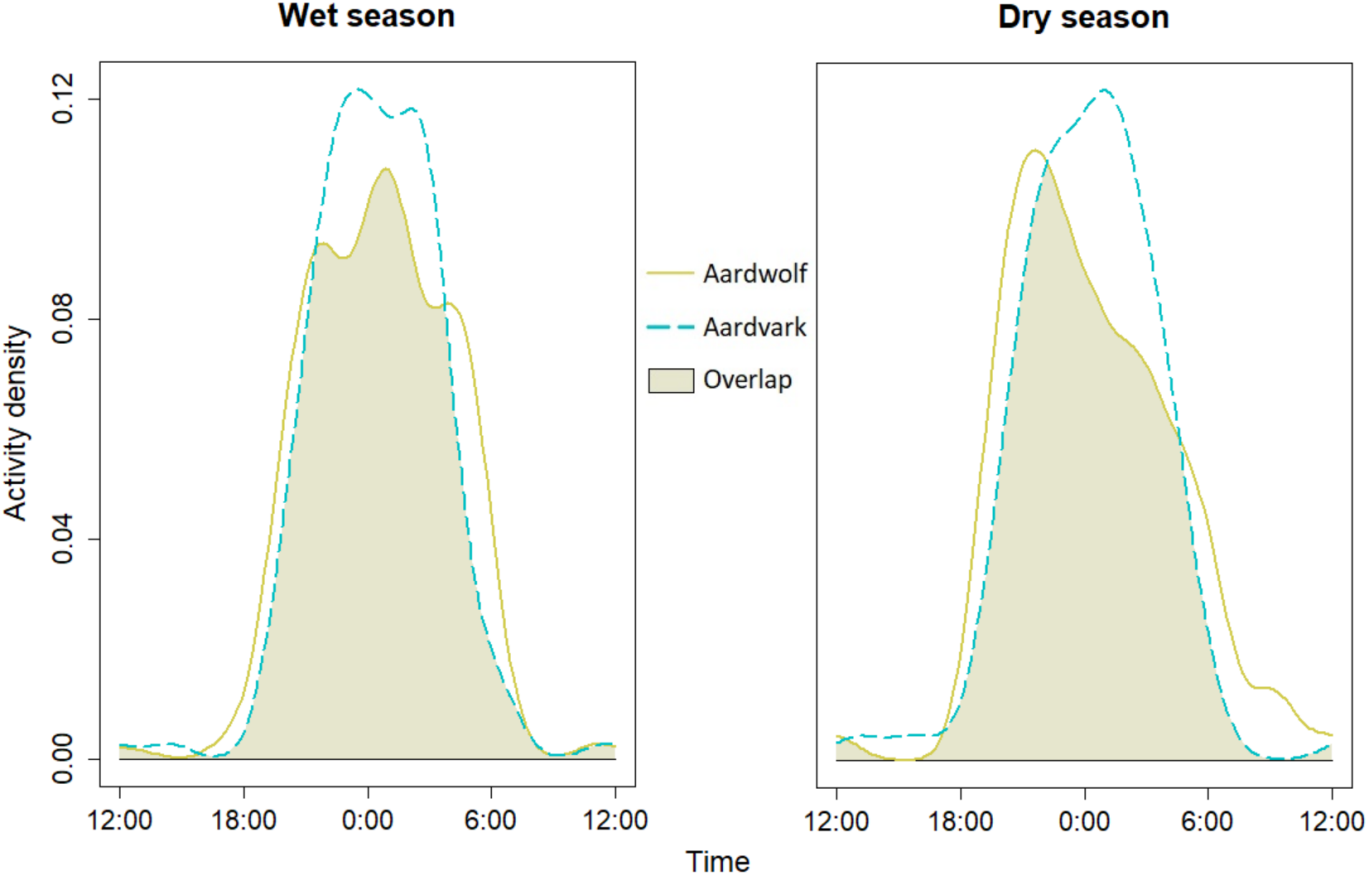
Density estimates of seasonal (wet = June–October, dry = November–May) diel activity of aardwolf (*Proteles cristata*) and aardvark (*Orycteropus after*), Serengeti National Park, Tanzania, 26 August 2016–30 June 2018.

Overlap in diel activity between aardwolves and aardvarks was high (Δ_4_ = 0.85, 95% CI = 0.77–0.93), and similar between seasons (wet: Δ_4_ = 0.87, 95%, CI = 0.80–0.93; dry: Δ_4_ = 0.83, 95% CI = 0.74–0.93) (Figure 4). Large overlaps in confidence intervals between seasonal models suggested year‐round consistency in diel activity overlap of aardwolves and aardvarks. The greatest overlap in diel activity occurred at night (18:00–6:00), with aardvark activity more frequent than aardwolf activity during 22:00–3:00. There appeared to be limited increases in diurnal activity during the dry season for both species, whereby aardwolves displayed increased morning activity (6:00–12:00), and aardvarks displayed increased afternoon activity (12:00–18:00).

## 4 DISCUSSION

We quantified the spatial and temporal overlap of aardvarks and aardwolves in southeastern Serengeti National Park, Tanzania. Results from the multispecies occupancy model supported our prediction of a positive relationship between the occupancy probabilities of aardwolves and aardvarks. Contrary to our predictions, we did not find evidence of spatial niche segregation through differential habitat use, or spatial niche overlap through similar responses to prey availability or predator avoidance. We found instead a positive relationship between occupancy probability and distance to water, a proxy for predator avoidance, for aardwolves only. We found no relationships between the occupancy probability of aardvarks and any of our covariates. Our prediction of high, year‐round overlap in the diel activity of aardwolves and aardvarks was supported.

Previous observations of aardwolves using burrows dug by aardvarks (Anderson, 2013) and of aardwolves feeding alongside aardvarks when the latter excavates termite mounds (Taylor & Skinner, 2000) corroborate our evidence for spatial niche overlap between these species. Aardwolf occurrence is restricted to open habitats because of their dependence on grass‐harvesting *Trinervitermes* termites (Anderson, 2013; Kruuk & Sands, 1972), while aardvarks occur in more diverse habitats, presumably due to their wider dietary niche including wood‐harvesting termites such as *Macrotermes* and *Odontotermes* (Taylor & Skinner, 2004). In the largely homogenous Serengeti grasslands, these genera of wood‐harvesting termites have adapted to harvest grass (Freymann et al., 2010), potentially increasing dietary niche overlap and by extension, spatial niche overlap between aardwolves and aardvarks.

We found high, year‐round temporal niche overlap in the diel activity of aardwolves and aardvarks, likely due to the nocturnality of their prey and thermoregulatory advantages of being active at night (Anderson, 2004; Weyer et al., 2020). Aardwolves and aardvarks in South Africa increased diurnal activity during winter when nocturnal prey is less active (Richardson, 1987; Taylor & Skinner, 2003), and during droughts (Rey et al., 2017; Weyer et al., 2020). Our analysis suggests limited increases in diurnal activity for both species during the dry season, potentially related to seasonal differences in prey activity (Materu et al., 2013). Year‐round stable temperatures in the Serengeti ecosystem (Metzger et al., 2015) may facilitate consistent foraging opportunities for aardwolves and aardvarks, in contrast to South Africa where aardwolves and aardvarks strongly increase diurnal activity during winter when termite activity was low (Rey et al., 2017; Richardson, 1987; Taylor & Skinner, 2003).

Overall, we found evidence for spatial and temporal niche overlap for aardwolves and aardvarks, congruent with research suggesting co‐occurrence of mammalian insectivores with similar diets is often driven by spatial and temporal activity patterns (Davis et al., 2018). Co‐occurring species that rely on the same, limited food sources may compete for access to these resources in space and time (Hardin, 1960), but aardwolves and aardvarks are myrmecophagous and may benefit from high food availability (Taylor & Skinner, 2004) reducing the potential for competition. High prey abundance could explain the prevalence of inter‐and intraspecific associative feeding, rather than competition for food, between myrmecophagous birds and mammals (e.g. Stenkewitz & Kamler, 2008; Taylor, 2013; Taylor & Skinner, 2000). Alternatively, subtle differences between species may explain coexistence without direct competition (Wiens, 1977). Aardwolves are morphologically adapted to consume termites from the surface (Anderson, 2013; Williams et al., 1997), whereas aardvarks have sharp claws to excavate mounds and an extensible tongue to extract prey from mound tunnels (Taylor et al., 2002). Aardwolves and aardvarks may avoid competition through fine‐scale spatial niche partitioning whereby aardvarks specialize on prey within mounds, and aardwolves on prey surrounding mounds. This idea is supported by reports of commensalism whereby aardwolves consume termites exiting mounds when excavated by aardvarks (Taylor & Skinner, 2003).

We found a positive relationship between aardwolf occupancy probabilities and distance from water, which could relate to spatial avoidance of large carnivores. Aardwolves and aardvarks largely do not rely on surface water and obtain water through prey consumption (Anderson, 2004; Taylor & Skinner, 2004), so it is unlikely their response to water source proximity is linked to physiological needs. Many herbivores depend on surface water, thus large carnivores preying on herbivores often hunt in close proximity to water (Constant et al., 2015; De Boer et al., 2010; Tagwireyi et al., 2020). Aardvarks in South Africa avoided areas close to water, potentially to avoid predation (Epps et al., 2021). Little is known about aardwolves and aardvarks as prey species, but they have few defences against large carnivores (Anderson, 2013; Taylor, 2013). Aardvarks could benefit from the many burrows they excavate that provide refuge from predators (Melton, 1976; Taylor & Skinner, 2003) while aardwolves may have only one or a few dens to use as refuge (Richardson, 1985). Aardwolves therefore may have fewer escape options from predators (Bothma & Walker, 1999), resulting in stronger spatial avoidance of large carnivores. However, further research is needed to definitively link proximity to water with predation risk for these species.

We note several limitations to our study. The model covariates we selected were based on studies from South Africa and Uganda, but the extent to which the ecological niche of aardwolves and aardvarks differs across their distribution is unknown. Our study area is characterized by limited variability in seasonal temperature differences, slope and proportional grass cover, which may be why no relation between these covariates and occupancy probabilities of aardwolves or aardvarks was found. Additionally, it is unknown whether termite mound density and slope on a 50‐m radius are accurate year‐round estimators of prey availability, particularly for aardvarks which elsewhere rely primarily on ants (Taylor et al., 2002; Willis et al., 1992). Similarly, our distance to water layer was imperfect due to consistent and complete datasets on ephemeral water sources being unavailable (Rich et al., 2017). Finally, our occupancy model assumed an individual present at a site occupied this site throughout each 70‐day period, which may not be the case due to mortality or individuals altering their space use, though we consider this unlikely due to the territoriality and longevity of aardwolves and aardvarks (Anderson, 2013; Taylor, 2013).

Aardwolves and aardvarks have an ecological role as termite consumers (Anderson, 2013), and aardvarks are considered ecosystem engineers through the excavation of burrows which benefit many vertebrate species (Whittington‐Jones et al., 2011). These burrows provide sleeping shelter for various small and medium‐sized mammals, and African wild dogs (*Lycaon pictus*) might use aardvark burrows to shelter young (Taylor, 2013). Aardvark burrows also contribute to local soil and vegetation diversity (Louw et al., 2017). While aardwolves and aardvarks are currently classified by the IUCN as species of least concern (Green, 2015; Taylor & Lehmann, 2015), a better understanding of their ecological niches can help identify current and future conservation issues including habitat loss (Green, 2015) and climate change (Rey et al., 2017). Finally, species interactions are primarily studied in the form of competitive interactions, but little is known regarding symbiotic relationships, so these results contribute to a better understanding of this understudied species interaction.

## AUTHOR CONTRIBUTIONS

**Merijn van den Bosch:** Conceptualization (lead); data curation (equal); formal analysis (lead); methodology (lead); visualization (lead); writing – original draft (lead); writing – review and editing (equal). **Kenneth F. Kellner:** Conceptualization (equal); data curation (equal); writing – review and editing (equal). **Imani Mkasanga:** Data curation (equal); project administration (equal); writing – review and editing (equal). **Stanslaus B. Mwampeta:** Data curation (equal); project administration (equal); writing – review and editing (equal). **Robert Fyumagwa:** Conceptualization (supporting); project administration (equal); writing – review and editing (equal). **Mariela G. Gantchoff:** Conceptualization (equal); writing – review and editing (equal). **Brent R. Patterson:** Conceptualization (equal); writing – review and editing (equal). **Jerrold L. Belant:** Conceptualization (equal); funding acquisition (equal); project administration (equal); supervision (lead); writing – review and editing (equal).

## CONFLICT OF INTEREST STATEMENT

The authors declare that they have no known competing financial interests or personal relationships that could have appeared to influence the work reported in this paper.

## Data Availability

Animal location data and code related to the analysis are available from Dryad through https://doi.org/10.5061/dryad.9kd51c5qb. This link is currently not live but will be after the paper is accepted for publication. Reviewers can access the dataset for peer review purposes through the following link: https://datadryad.org/stash/share/nDrQ5zc37KII8eJ1UNJJ3DDYden0zGKbuTtKMrh9GY0.
